# Impact of Vancomycin Resistance on 30-Day Mortality in Solid Organ Transplant Recipients with *Enterococcus faecium* Bloodstream Infections: A Retrospective Cohort Analysis

**DOI:** 10.3390/antibiotics15020119

**Published:** 2026-01-26

**Authors:** Maria Mazzitelli, Alberto Enrico Maraolo, Umberto Barbieri, Vincenzo Scaglione, Lolita Sasset, Lucrezia Furian, Umberto Cillo, Gino Gerosa, Monica Loy, Emanuele Cozzi, Patrizia Burra, Federico Rea, Annamaria Cattelan

**Affiliations:** 1 Infectious and Tropical Diseases Unit, Padua University Hospital, 35128 Padua, Italy; 2 Dipartimento di Scienze Mediche e Chirurgiche, Fondazione Policlinico Universitario Agostino Gemelli IRCCS, 00168 Rome, Italy; 3 Dipartimento di Sicurezza e Bioetica, Sezione di Malattie Infettive, Università Cattolica del Sacro Cuore, 00168 Rome, Italy; 4 Section of Infectious Diseases, Department of Clinical Medicine and Surgery, University of Naples “Federico II”, 80138 Naples, Italy; 5 Kidney and Pancreas Transplantation Unit, Department of Surgical Gastroenterological and Oncological Sciences, Padua University Hospital, 35128 Padua, Italy; 6 Hepatopancreatobiliary Surgery and Liver Transplantation, Padua University Hospital, 35128 Padua, Italy; 7 Cardiac Surgery Unit, Department of Cardiac, Thoracic, Vascular Sciences and Public Health, University of Padua, 35122 Padua, Italy; 8 Department of Cardiac, Thoracic, Vascular Sciences, and Public Health, Padua University Hospital, 35128 Padua, Italy; 9 Transplant Immunology Unit, Department of Cardiac, Thoracic and Vascular Sciences, University of Padua, Hospital-Ospedale Giustinianeo, 35121 Padua, Italy; 10 Department of Gastroenterology, University of Padua, 35122 Padua, Italy; 11 Thoracic Surgery Unit, Department of Cardiac, Thoracic, Vascular Sciences and Public Health, University of Padua, 35122 Padua, Italy

**Keywords:** *Enterococcus faecium*, bacteraemia, bloodstream infections, mortality, predictors, solid organ transplant, transplant recipients

## Abstract

**Background:** *Enterococcus faecium* bloodstream infections (EF-BSI) cause significant morbidity and mortality in solid organ transplant (SOT) recipients, with the role of vancomycin resistance (VR) remaining controversial as an independent driver. **Methods:** This was a retrospective cohort study including SOT recipients with EF-BSI at our institution from 2019 to 2023. We used Cox proportional hazards regression to identify predictors of 30-day all-cause mortality. A time-dependent covariate was used to model the effects of receiving targeted, effective antibiotic therapy. **Results:** A total of 79 patients were included (26.6%, with VR). The overall 30-day mortality was 12.7% (10/79). In univariable analysis, septic shock (Hazard Ratio, HR: 17.1, 95% CI: 3.64–80.8, *p* < 0.001), the need for Continuous Venovenous Hemofiltration (HR: 6.40, 95% CI: 1.85–22.1, *p* = 0.003), and a Pitt Bacteremia Score ≥ 2 (HR: 5.17, 95%CI: 1.10–24.3, *p* = 0.038) were associated with increased mortality, while source control was protective (HR: 0.20, 95% CI: 0.05–0.76, *p* = 0.018). In the final multivariable model, only septic shock remained an independent predictor of 30-day mortality (HR: 11.4, 95% CI: 1.63–79.5, *p* = 0.014). VR was not significantly associated with mortality, though the confidence interval was wide and included clinically meaningful effects (HR: 2.07, 95% CI: 0.40–10.6, *p* = 0.4). **Conclusions:** In SOT recipients with EF-BSI, 30-day mortality is overwhelmingly driven by the host’s physiological response, manifested as septic shock, rather than the VR profile of the pathogen. The early recognition of severe sepsis/septic shock and the aggressive implementation of supportive care and source control measures in this setting are crucial.

## 1. Introduction

*Enterococcus faecium* (*E. faecium*) emerged as a threat pathogen among solid organ transplant (SOT) recipients [1]. This patient population is particularly vulnerable due to a confluence of risk factors, including profound immunosuppression, prolonged and recurrent hospitalizations, a frequent use of indwelling medical devices, and an extensive exposure to broad-spectrum antimicrobials [2]. These conditions create a fertile environment for colonization and subsequent infection with multidrug-resistant organisms [3].

*E. faecium* bloodstream infections (BSIs) in SOT recipients are associated with significant morbidity, including persistent or recurrent bacteraemia, prolonged hospital stays, and unacceptably high mortality rates [2].

A central controversy in the management of such infections is the clinical significance of vancomycin resistance (VR). Historically, numerous studies and several meta-analyses have indeed reported an association between VR *Enterococcus* BSI and increased mortality compared to infections caused by vancomycin-susceptible *Enterococcus* (VSE) [4,5]. However, many of these analyses were conducted before the widespread availability of effective anti-VRE therapies, such as linezolid (2000) and daptomycin (2003). More recent and methodologically rigorous studies controlling for critical confounding variables (such as the severity of underlying illness) have increasingly challenged this association, suggesting that VR may not be an independent driver of mortality [6]. This has given rise to the hypothesis that VRE is not an inherently more virulent pathogen but rather serves as a marker for a more complex and critically ill host—one whose extensive healthcare exposures have selected for colonization with a resistant organism [7,8].

The specific impact of VR on outcomes in the high-risk SOT population remains inadequately defined in the contemporary therapeutic era. Therefore, the primary objective of this study was to identify the predictors of 30-day mortality among SOT recipients with *E. faecium* BSIs and to specifically determine whether VR is an independent risk factor after adjusting for clinical severity and therapeutic interventions.

## 2. Results

### 2.1. Cohort Characteristics

A total of 79 patients, all Caucasians, were included (58, 73%, with VSE infections and 21, 27%, with VRE); see Table 1. The median age was 64 years, and the majority of patients were female (70.9%), with no significant age or sex differences between VSE and VRE cohorts (*p* = 0.07 and 0.73, respectively). The distribution of transplant types differed between groups (overall *p* = 0.03), mostly for kidney transplant recipients, accounting for a larger proportion of the VRE group (33.3% of VRE cases vs. 8.6% of VSE cases), and liver transplant recipients, who predominated in the VSE group (72.4% of VSE vs. 42.9% of VRE). The comorbidity burden was high in both groups (median Charlson Comorbidity Index = 5 in each), and the rates of underlying conditions such as diabetes, cardiovascular disease, or liver cirrhosis did not significantly differ (all *p* > 0.1). Nearly all infections were hospital-acquired (nosocomial in ~80–86% of cases) and occurred early post-transplant (Table 1).

Approximately 20% of patients in each group were in the intensive care unit (ICU) at BSI onset. Importantly, the clinical severity at presentation was similar: nearly all patients fulfilled sepsis criteria (by design, 99% had sepsis), and a subset presented with septic shock (22.4% VSE vs. 14.3% VRE, *p* = 0.63). The median Pitt score was 1 in both groups (IQR: 1–3 in VSE vs. 1–2 in VRE), reflecting a range of illness severity but no significant inter-group differences.

The presumed sources of *E. faecium* BSI were predominantly intra-abdominal infections (e.g., abscess, anastomotic leak, or cholangitis) in both cohorts (65.5% VSE vs. 52.4% VRE had intra-abdominal source; *p* = 0.42). Central line-associated bloodstream infection was identified in ~25% of cases (similar between groups). Notably, urinary tract sources were significantly more frequent in the VRE group (19.0% vs. 1.7% in VSE, *p* = 0.023). Four VRE BSI cases were attributed to urinary tract infection (often in kidney transplant patients with indwelling catheters), compared to only one case in the VSE group. Polymicrobial infection (concurrent isolation of other bacterial or fungal pathogens) occurred in roughly one-quarter of cases (24% VSE, 19% VRE), with no significant difference (commonly co-isolated organisms included *E. coli*, *Klebsiella*, *Candida* spp., among others in polymicrobial infections). Persistent bacteremia was observed in 13 (16.5%) cases, more commonly in the VSE group.

All details on *E. faecium* resistance profiles and antimicrobial therapy are reported in Supplementary Table S1. All patients received some empiric antibiotic therapy at the onset of sepsis. However, the initial regimens were less likely to be effective against *E. faecium* in the VRE group. Only 9.5% of VRE cases (2/21) had an appropriate empiric coverage, compared to 34.5% of VSE cases (20/58), a trend toward significance (*p* = 0.057). Indeed, 29% of VSE patients received monotherapy with a glycopeptide (vancomycin or teicoplanin) as a definitive therapy, whereas none of the VRE patients did (since their isolates were vancomycin-resistant; *p* = 0.013). Conversely, linezolid use was more frequent in VRE cases (38% received linezolid monotherapy vs. 10% of VSE, *p* = 0.012). The median time to the initiation of an active targeted antibiotic (after culture collection) was 2 days in both groups (IQR: 0–3 days for VSE, 1–4 days for VRE, *p* = 0.15), indicating that, once the pathogen and susceptibilities were known, therapy was adjusted promptly. Nearly all patients (96–97%) ultimately received targeted therapy appropriate for the isolate, except for two patients (one VSE, one VRE) who died very early before effective therapy could be delivered. Other management aspects were similar: an ID specialist was consulted in 81% of cases (both groups), and an attempt at source control (such as surgical intervention or device removal) was made in 66% of cases in both groups (Table 1). Trans-thoracic echocardiography (TTE) was performed in 72% of patients to screen for endocarditis, with no difference between VRE and VSE groups, while trans-oesophageal echocardiography (TEE) was performed in a minority (10% VSE vs. 24% VRE, *p* = 0.25), often in those with persistent bacteraemia or high clinical suspicion. No definite endocarditis was diagnosed in this cohort. The overall 30-day mortality and relapse rate were 12.7% (10/79) and 29.1% (23/79), respectively.

### 2.2. Univariable and Multivariable Analysis

We analyzed each clinical factor’s association with 30-day mortality in a Cox model. At univariable analysis (Table 2), septic shock emerged as the strongest predictor of death: patients who presented in shock had an HR of 17.1 for 30-day mortality (95% CI: 3.64–80.8, *p* < 0.001).

The need for acute renal replacement (CVVH dialysis) was also significantly associated with mortality (HR: 6.40, 95% CI: 1.85–22.1, *p* = 0.003), reflecting that patients with fulminant multi-organ failure fared poorly. Not achieving source control of the infection was another important factor—if the infection focus could not be removed or drained, outcomes worsened: the presence of source control was protective with an HR of 0.20, meaning the absence of source control carried an approximate 5-fold higher hazard of death (*p* = 0.018). Higher initial Pitt bacteraemia scores correlated with mortality: each 1-point increase in the Pitt score conferred HR 2.02 (*p* = 0.001), and having a Pitt score ≥ 2 was associated with HR 5.17 (*p* = 0.038). Other markers of illness such as ICU admission and mechanical ventilation had non-significant trends toward higher risk. Interestingly, the VR status did not significantly affect mortality in the unadjusted analysis (HR: 1.98, 95% CI: 0.56–7.03, *p* = 0.30).

**Figure 1:**
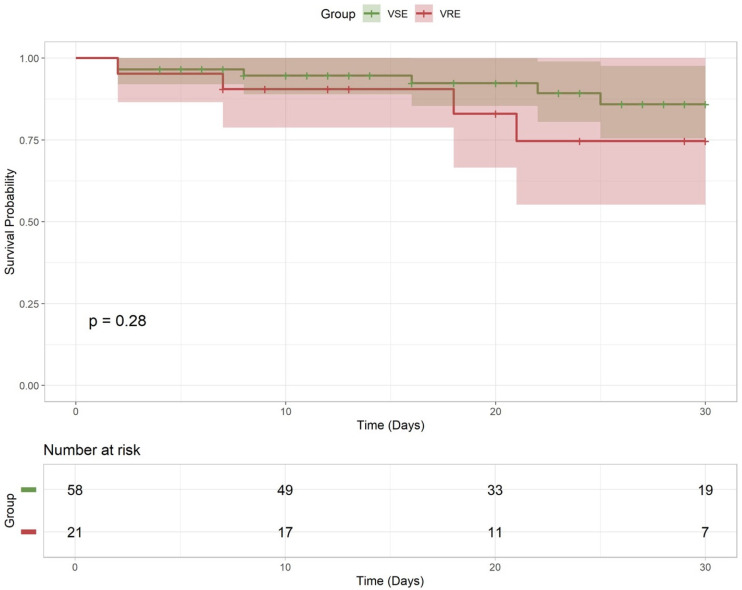
The 30-day survival in SOT patients with *E. faecium* BSI. Legend to Figure 1: Kaplan–Meier showing 30-day mortality.

The Kaplan–Meier analysis (Figure 1) of 30-day survival likewise showed no significant divergence between VRE and VSE (*p* = 0.38).

Patients who empirically received an appropriate antibiotic showed a trend toward better survival (HR: 0.24, CI up to 1.86), but this did not reach statistical significance (*p* = 0.20). The time-dependent covariate for effective therapy indicated a strong protective point estimate (HR: 0.18, suggesting an 82% reduction in hazard once effective therapy was started), but, given the wide CIs (0.01–2.39) and *p* = 0.20, this did not meet significance. Performing a TTE had a borderline association with lower mortality (HR: 0.35, *p* = 0.10), possibly reflecting that patients who survived longer were more likely to undergo thorough evaluation (or that those who died too quickly never received an echocardiogram). Other factors such as age, sex, transplant organ type, comorbidities, polymicrobial infection, or ID consultation were not significantly linked to 30-day outcomes in the univariate analysis (all *p* > 0.2).

The multivariable Cox model included septic shock, CVVH requirement, source control, the performance of TTE, Pitt score category (at least 2 points), VR status, and the time-dependent effective therapy variable (these were the factors meeting inclusion criteria as described). The results of the adjusted analysis are shown in Table 3.

Septic shock remained the sole independent predictor of 30-day mortality, with an aHR of 11.4 (95% CI: 1.63–79.5, *p* = 0.014). This underscores the overwhelming impact of shock on outcomes. In contrast, after adjustment, vancomycin-resistant infection still did not significantly affect mortality (aHR: 2.07, 95% CI 0.40–10.6, *p* = 0.40). The point estimate was above 1, but the confidence interval was wide, reflecting the small number of events. Similarly, receiving effective antibiotic therapy was associated with an aHR of 0.31 (suggesting benefits), but this was not statistically significant (95% CI: 0.02–4.11, *p* = 0.40). None of the other covariates retained statistical significance in the multivariable model: the need for CVVH had an aHR: 2.0 (*p* = 0.4), the lack of TTE had an aHR: 2.0 (*p* = 0.4 for TTE performed being protective), a high Pitt score had an HR: 1.33 (*p* = 0.8), and source control had an aHR: 0.54 (*p* = 0.5).

As explained in the methods, we conducted a sensitivity analysis rather than a post hoc power analysis. The observed 30-day mortality rates were 10.3% (6/58) in the VSE group and 19.0% (4/21) in the VRE group. This corresponds to an observed absolute risk difference of 8.7%. The sensitivity analysis indicated that, given the baseline mortality of 10.3% in the VSE group and the sample size of the VRE group (n = 21), the study was only powered to detect a mortality rate of approximately 45% or higher in the VRE group (an absolute difference of >34 percentage points). The 95% CI for the observed risk difference of 8.7% was wide, ranging from −9.8% to +27.2%.

## 3. Discussion

The main finding of this study is that, in a cohort of SOT recipients with *E. faecium* BSI, the development of septic shock was the single most powerful and independent predictor of 30-day mortality. After rigorously accounting for illness severity and other clinical factors, the VR profile of the infecting organism was not associated with an adverse outcome. This result contributes to a growing body of evidence suggesting that the association between VRE and mortality observed in previous studies may have been driven by confounding variables, in contrast with previous data [6,7,8].

SOT represents a risk factor for VRE BSI, but the VR status per se is not a driver of mortality, although, in other settings, such as in hematological patient, there is opposing evidence [9,10].

At any rate, our findings support a conceptual shift in the understanding of VRE, at least in the context of SOT. Rather than viewing VRE as an intrinsically hyper-virulent pathogen, it may be more accurately considered an epiphenomenon of a specific, high-risk host. The data show that VRE BSI was significantly more common in kidney transplant recipients and those with a urinary source of infection [2]. This patient phenotype is characterized by frequent and prolonged healthcare contact, chronic catheterization, and repeated courses of antibiotics—all established risk factors that select for gastrointestinal colonization with VRE [2]. In this context, the organism’s resistance pattern is a consequence of the host’s prior medical history and exposures, rather than being the primary factor influencing outcomes after an invasive infection [11,12,13]. Ultimately, the outcome is likely driven by the host’s capacity to withstand the physiological insult of BSI [14,15].

Multiple studies demonstrate that vancomycin-resistant *E. faecium* (VREfm) isolates frequently harbour putative virulence genes such as esp, gelE, and asa1, but the presence of these genes does not consistently correlate with an increased clinical virulence or mortality. Genome-wide association studies have not identified a distinct virulence profile linked specifically to VR [16].

In line with previous Italian and international experiences, our findings confirm that vancomycin resistance per se does not independently predict early mortality in *E. faecium* bloodstream infections among solid organ transplant recipients once disease severity and timely active therapy are accounted for. Indeed, both Rinaldi et al. and Dal Monte et al. demonstrated similar results in large multicentric cohorts, where vancomycin resistance lost its prognostic significance after adjustments for the SOFA score, infection source, and delay to appropriate treatment, underscoring the dominant role of the clinical condition at onset and adequacy of source control [6,17]. Comparable conclusions were reached by Rothe et al., who, in a mixed cohort of *Enterococcus* bacteraemia, found that species (*E. faecium*) rather than resistance phenotype determined long-term outcomes [18]. Conversely, Adelman et al. identified vancomycin resistance as an independent predictor of 90-day mortality in a broader SOT population with any-pathogen bloodstream infection, possibly reflecting the cumulative effects of late complications, persistent colonization, and comorbid burden in this fragile population [19]. Earlier observations from Dubberke et al. in hematologic patients similarly suggested that VRE bacteraemia clusters in the most severely ill, functioning more as a marker of underlying vulnerability than as a direct virulence determinant [20]. VSE and VRE patients in our cohort had a comparable clinical severity at presentation—identical median Pitt scores (1 in both groups), similar rates of septic shock (22.4% vs. 14.3%, *p* = 0.63), and similar ICU admission rates (20.7% vs. 19.0%). If VR were intrinsically driving worse outcomes through increased virulence, we would expect VRE patients to present with more severe disease, as well as a loss of septic shock’s independent significance when adjusting for VR. Importantly, VRE became among the leading cause of BSIs in patients who undergo allogenic stem cell transplant, especially in the early phase [21], as well as liver transplant recipients, in whom VRE colonization (unfortunately not investigated in the present study) was associated with a significant risk of infection [22]. A recent multicentre analysis showed that people who received solid organ transplant have a significantly higher risk for 30-day mortality [17], while showing in the overall population that follow-up blood culture and source control were associated with a favourable outcome.

Among the 10 deaths, 9 occurred in patients with septic shock (56% of all shock patients died). The overwhelming impact of septic shock, with a more than 11-fold increased hazard of death, underscores that the host’s dysregulated inflammatory response is the final common pathway to mortality [2]. This aligns with the modern understanding of sepsis, where organ dysfunction resulting from the host response, rather than the direct action of the microbe, is the principal driver of death [23]. Other significant univariable predictors, such as the need for CVVH and a high Pitt score, lost their independent predictive power in the multivariable model. Of course, the small sample size may have influenced this loss of predictive power, but these variables, besides being potentially distinct risk factors, can be considered as expressions integral to the syndrome of septic shock. Specifically, CVVH is a treatment for acute kidney injury associated with shock, while a high Pitt score reflects the multi-organ dysfunction that defines it [24,25].

We evaluated the Pitt score as both a continuous and a categorical variable. The decision to categorize the score was based on results from the VENOUS cohort, which associated a score of ≥2 with mortality [26]. The categorical variable was ultimately used in the multivariable model because it produced a higher HR (and lower *p*-value) in the univariable analysis.

The strong protective effect of source control in the univariable analysis (HR 0.20) highlights its fundamental importance in the management of BSI, a principle that is well established [17]. Although it did not retain statistical significance in the final multivariable model, this is likely attributable to the limited statistical power of the study and the overwhelming predictive strength of septic shock, with which it is clinically intertwined. The VRE group’s trend toward less frequent appropriate empirical therapy did not translate into a higher adjusted mortality, reinforcing the conclusion that the severity of the initial septic response frequently determines the host’s clinical trajectory; a state that may be established before a 24 to 48 h delay in optimal antibiotic therapy can be overcome [27].

We also found that early effective antibiotic therapy (modelled as a time-dependent covariate) was not clearly associated with a reduced mortality in this study. This result is surprising, as delays in appropriate therapy are traditionally linked to worse outcomes in sepsis [28]. However, while the point estimate suggests a potential protective effect, the wide confidence interval reflects sparse outcome events and limited statistical power. This imprecision is further compounded by the fact that nearly all survivors received effective therapy within 48–72 h, providing a minimal variability in exposure for analysis. There may have been insufficient power to detect the benefit of shaving off several hours in therapy initiation. Additionally, in the context of extremely rapid fulminant infections (e.g., patients who died within 1–2 days), even the best-case scenario of prompt appropriate antibiotics might not have averted the outcome due to irreversible shock and organ failure. In our cohort, all patients who died early were in septic shock at presentation, and some actually had appropriate empiric coverage (yet still succumbed). Thus, our findings should not be interpreted as implying that appropriate therapy is unimportant, but rather that, when severe sepsis occurs, other interventions (e.g., aggressive resuscitation, source control) may play a more decisive role in survival [29]. The data underscore the critical importance of the early recognition and management of sepsis in transplant patients. For clinicians, this means that, while choosing empiric antibiotics that cover VRE may be a consideration (especially in known high-risk patients), the highest priority should be patient stabilization and source control.

We acknowledge that the study period (January 2019–December 2023) partially overlaps with the COVID-19 pandemic, which could theoretically influence both infection epidemiology and clinical outcomes in transplant recipients. To address this concern, we carefully reviewed the temporal distribution of cases and outcomes across the study years. The number of *E. faecium* bloodstream infections did not show a significant deviation during 2020–2021 compared with pre- and post-pandemic years, and no clustering of vancomycin-resistant isolates or excess mortality was observed in those years. Importantly, our centre maintained continuous transplant and infectious disease services and major activities throughout the pandemic, with only limited elective activity reductions (such as outpatient services). Also, targeted protocols and recommendations have been developed to reduce the risk of COVID-19 transmission and to guide the safe resumption of living-donor kidney transplant programmes, including strategies such as transitioning donor and recipient evaluations and follow-up visits to telehealth [30].

Our study has several limitations. The sample size is modest and drawn from a specific population of SOT patients with *E. faecium* BSIs, limiting the generalizability to all transplant settings. The number of outcome events was small, constraining the multivariate analysis and leading to wide confidence intervals. Given the low power, the study may have failed to detect a true, albeit smaller, effect of VR or other variables on mortality (type II error).

The precision analysis mandates a cautious interpretation of this finding as well. The wide 95% confidence interval indicates that our data are compatible with true effect sizes ranging from a 10% reduction in mortality to a clinically substantial 27% increase in the impact of VR. Furthermore, the sensitivity analysis reveals that the study was underpowered to detect moderate increases in risk; it was only capable of statistically identifying catastrophic effect sizes (e.g., a tripling of the mortality rate). Therefore, while we did not observe VRE to be an independent driver of mortality in this specific cohort, the possibility of a type II error remains, and we cannot definitively rule out VRE as a risk factor for adverse outcomes in SOT.

There may be residual confounders we could not fully adjust for, such as differences in clinical management or variations between transplant centres. The analysis focused on 30-day mortality; it is possible that VR could have more subtle impacts on longer-term outcomes (90-day mortality, graft function, or recurrence rates) that we did not capture. Additionally, we did not formally assess the impact on length of stay or costs, which are known to be higher with VRE infections. Sparse data and limited events per variable in our analysis raise the possibility of type II errors (failing to detect a real effect). Therefore, larger, multicentre studies or pooled analyses would be valuable to clarify the true impact of VRE on mortality and long-term outcomes like graft survival. Despite these limitations, our study provides insight into acute phase outcomes and highlights key factors like septic shock and source control in a transplant cohort.

## 4. Materials and Methods

### 4.1. Study Design and Population

A retrospective cohort study was conducted at a large, tertiary-care transplant centre (Azienda Ospedale Università di Padova, Veneto, Italy). The study population included all adult (≥18 years) recipients of an SOT (liver, kidney, heart, lung, pancreas, or multi-organ) who had at least one positive blood culture for *E. faecium* from January 2019 to December 2023. The index date was defined as the collection date of the first positive blood culture. In our setting, during the study period, the pattern of resistance of *E. faecium* detected on blood cultures was as follows: a high level of resistance for aminoglycosides in 51.3% cases, vancomycin resistance in 25.2% cases, and linezolid resistance in 1.1% cases.

As a retrospective observational study, the sample size was determined by the actual number of eligible cases during the study period rather than by prospective power calculations. All consecutive SOT recipients with EF-BSI in the above-mentioned timeframe were included to maximize case ascertainment within a contemporary treatment era.

### 4.2. Outcome Measure

The primary outcome was the all-cause mortality within 30 days of BSI onset. Mortality at 30 days was determined via medical records and transplant follow-up databases. Patients alive at 30 days were censored at that time for survival analyses. Secondary outcomes (described descriptively) included persistent bacteremia (defined as positive blood cultures > 3 days from BSI onset) and 90-day infection relapse. Appropriate analyses were also used to assess variables associated with the risk of death.

### 4.3. Data Collection and Definitions

Data were extracted from the medical health record. Variables collected included demographics, comorbidities (summarized by the Charlson Comorbidity Index), and transplant-specific details. Microbiological data included species identification, vancomycin susceptibility status (VRE vs. VSE), and the presence of co-pathogens in the index blood culture (polymicrobial BSI). In particular, blood cultures were processed in the institutional clinical microbiology laboratory according to routine diagnostic procedures. Positive blood cultures were sub-cultured, and isolates were identified at the species level as *E. faecium* using standard laboratory identification methods (e.g., automated identification and/or MALDI-TOF MS, according to local availability). Antimicrobial susceptibility testing was performed on the index bloodstream isolate using routine phenotypic AST methods (automated MIC-based system and/or reference MIC methods where required by laboratory policy). Susceptibility categories were interpreted according to the current EUCAST breakpoints in force at the time of testing. Vancomycin resistance was defined phenotypically based on the vancomycin MIC and corresponding categorical interpretation (VRE vs. VSE). In cases of borderline/discordant results, vancomycin MIC determination was repeated and/or confirmed using an alternative MIC method (e.g., gradient diffusion or reference microdilution, per laboratory practice). Clinical severity at the onset of BSI was assessed using the Pitt bacteraemia score and the presence of sepsis or septic shock, as defined by the Sepsis-3 criteria. Therapeutic interventions, including the presence of central venous catheters (CVCs), requirements for Continuous Venovenous Hemofiltration (CVVH), and the achievement of source control, were documented. Source control was defined as the definitive drainage of abscesses, removal of infected hardware, or surgical debridement of infected tissue. Antibiotic regimens were recorded, and the following definitions were used:

○ Empirical therapy: initial antibiotics before culture results;
○ Appropriate empirical therapy: empirical regimen with in vitro activity against final isolate, initiated ≤24 h from culture collection;
○ Targeted therapy: antibiotic regimen adjusted based on susceptibility results;
○ Effective therapy: any antibiotic regimen (empirical or targeted) with documented in vitro activity against the isolate.

### 4.4. Statistical Analysis

Data completeness was assessed for all variables. No missingness was detected for the described variables. Missing data concerned covariates of minor interest for the present study (e.g., daptomycin resistance), so they were not taken into account. Baseline characteristics were summarized for VSE vs. VRE groups. Categorical variables were compared using Fisher’s exact test or chi-square, and continuous variables using the Wilcoxon rank-sum test, as appropriate, with *p* < 0.05 considered significant. Survival probabilities were estimated using the Kaplan–Meier method and compared between groups using the log-rank test. We constructed Cox proportional hazards models to evaluate associations with 30-day mortality. To account for time-varying exposure to effective therapy, we incorporated a time-dependent covariate for the initiation of effective antibiotic therapy: patients contributed person-time to the “unexposed” category from BSI onset until the initiation of an antibiotic with documented in vitro activity against the isolate, after which they contributed person-time to the “exposed” category. Time zero was defined as the date of index blood culture collection. This method mitigates immortal time bias since patients who died before receiving effective therapy contribute person-time in the unexposed category [31]. Covariates with *p* < 0.05 in univariable screening were considered for the multivariable model in order to avoid overfitting, alongside covariates deemed clinically important (notably, VR status and the time-dependent effective therapy variable were forced into the model regardless of *p*-value). A backward elimination approach was not used; instead, we built the final multivariable Cox model including all selected covariates, given the limited number of outcome events. The proportional hazards assumption was checked by examining Schoenfeld residuals.

To assess the statistical capacity of the study given the fixed sample size, we performed a sensitivity analysis rather than a post hoc power analysis, in accordance with current statistical recommendations. We calculated the minimum detectable effect size for 30-day mortality between the VSE and VRE groups, assuming 80% power and a two-sided alpha of 0.05. Additionally, to evaluate the precision of the non-significant finding, we calculated the 95% CI for the absolute risk difference in mortality. This approach allows for the interpretation of the range of plausible effect sizes compatible with the observed data, distinguishing between a true negative result and an inconclusive one due to limited precision [32]. Results are reported as Hazard Ratios (HRs) with 95% confidence intervals (CIs); adjusted HRs (aHRs) ensued from multivariable regression. Statistical analyses were conducted using R (version 4.2) and the pwr, survival, and survminer packages.

## 5. Conclusions

In conclusion, in SOT recipients with *E. faecium* BSI, 30-day mortality seems to be a function of host-related factors that culminate in the syndrome of septic shock, rather than the VR of the infecting organism.

Within the limitations of our sample size and event rate, VR status did not demonstrate a statistically significant independent association with mortality. However, the wide confidence intervals preclude the definitive exclusion of a clinically meaningful effect. Infections by VRE may serve primarily as a marker of patients with complex medical histories and extensive healthcare exposures rather than as an independent determinant of mortality, though larger studies are needed to confirm this hypothesis with adequate precision.

These findings may emphasize the importance of clinical efforts focusing on the early recognition and aggressive management of sepsis, including hemodynamic support, and the timely implementation of source control measures to improve outcomes in this highly vulnerable population.

## Figures and Tables

**Table 1:** Features of the study cohort, overall and by vancomycin susceptibility.

Characteristics	Overall, n = 79 (%)	VSE, n = 58	VRE, n = 21	*p* Value
Sex, female, n (%)	56 (70.9)	40 (68.9)	16 (76.2)	0.731
Age, years, median (IQR)	64 (55–69)	62.5 (55–68)	66 (60–71)	0.072
Charlson Comorbidity Index, median (IQR)	5 (3–7)	5 (3–7)	5 (4–5)	0.814
Diabetes mellitus, n (%)	24 (30.4)	17 (29.3)	7 (33.3)	0.947
Hypertension, n (%)	34 (43.0)	22 (37.9)	12 (57.1)	0.205
Obesity, n (%)	7 (8.9)	6 (10.3)	1 (4.8)	0.746
Chronic obstructive pulmonary disease, n (%)	7 (8.9)	5 (8.6)	2 (9.5)	1.000
Ischemic heart disease, n (%)	20 (25.3)	14 (24.1)	6 (28.6)	0.914
Hematological cancer, n (%)	3 (3.8)	3 (5.2)	0 (0.0)	0.692
Solid cancer, n (%)	22 (27.8)	19 (32.8)	3 (14.3)	0.182
Liver cirrhosis, n (%)	35 (44.3)	27 (46.6)	8 (38.1)	0.680
Asplenia, n (%)	2 (2.5)	2 (3.4)	0 (0.0)	0.959
HIV, n (%)	1 (1.3)	1 (1.7)	0 (0.0)	1.000
Transplant type, n (%)				0.032
Heart	8 (10.1)	5 (8.6)	3 (14.3)	
Heart–kidney	1 (1.3)	1 (1.7)	0 (0.0)	
Kidney	12 (15.2)	5 (8.6)	7 (33.3)	
Kidney–pancreas	1 (1.3)	0 (0.0)	1 (4.8)	
Liver	51 (64.6)	42 (72.4)	9 (42.9)	
Lung	6 (7.6)	5 (8.6)	1 (4.8)	
Admission in the previous 3 months, n (%)	37 (46.8)	28 (48.3)	9 (42.9)	0.864
Admission in the previous 12 months, n (%)	57 (72.2)	41 (70.7)	16 (76.2)	0.843
Surgery 60 days before admission, n (%)	55 (69.6)	39 (67.2)	16 (76.2)	0.626
Medical ward admission, n (%)	31 (39.2)	26 (44.8)	5 (23.8)	0.153
Surgical ward admission, n (%)	32 (40.5)	20 (34.5)	12 (57.1)	0.120
ICU admission, n (%)	16 (20.3)	12 (20.7)	4 (19.0)	1.000
Communitarian infection, n (%)	15 (19.0)	12 (20.7)	3 (14.3)	0.752
Nosocomial infection, n (%)	64 (81.0)	46 (79.3)	18 (85.7)	0.752
Septic shock, n (%)	16 (20.3)	13 (22.4)	3 (14.3)	0.633
Pitt score, median (IQR)	1 (1–3)	1 (1–3)	1 (1–2)	0.535
Pitt score ≥ 2, n (%)	35 (44.3)	27 (46.6)	8 (38.1)	0.680
Polymicrobial, n (%)	18 (22.8)	14 (24.1)	4 (19.0)	0.863
Primary infection, n (%)	4 (5.1)	3 (5.2)	1 (4.8)	1.000
IAI, n (%)	49 (62.0)	38 (65.5)	11 (52.4)	0.423
CVC/intravascular device related, n (%)	21 (26.6)	16 (27.6)	5 (23.8)	0.962
Urinary tract infection, n (%)	5 (6.3)	1 (1.7)	4 (19.0)	0.023
Total parenteral nutrition, n (%)	39 (49.4)	26 (44.8)	13 (61.9)	0.277
Urinary catheter, n (%)	57 (72.2)	39 (67.2)	18 (85.7)	0.182
Gram negative rectal colonization, n (%)	8 (10.1)	6 (10.3)	2 (9.5)	1.000
CVC, n (%)	58 (73.4)	41 (70.7)	17 (81)	0.533
CVVH, n (%)	11 (13.9)	7 (12.1)	4 (19)	0.672
Neutropenia, n (%)	1 (1.3)	1 (1.7)	0 (0.0)	1.000
Serum creatinine, mmol/L, median (IQR)	84 (59–131)	78 (55–126)	98 (75–287)	0.089
eGFR, ml/min, median (IQR)	82 (45–107)	88 (46–110)	70 (14–95)	0.135
C-Reactive Protein, mg/dL, median (IQR)	77.00 (31–110)	72 (32–100)	100 (20–130)	0.415
Procalcitonin, ng/mL, median (IQR)	1.05 (0.05–3.45)	1.12 (0.19, 3.58]	0.13 (0.04–2.51)	0.223
Daptomycin dosage, mg/kg, n (%)				0.725
0	56 (70.9)	41 (70.7)	15 (71.4)	
6	5 (6.3)	4 (6.9)	1 (4.8)	
8	10 (12.7)	6 (10.3)	4 (19.0)	
10	6 (7.6)	5 (8.6)	1 (4.8)	
12	2 (2.5)	2 (3.4)	0 (0.0)	
Source control, n (%)	52 (65.8)	38 (65.5)	14 (66.7)	1.000
ID consultation, n (%)	64 (81)	47 (81.0)	17 (81.0)	1.000
Drainage, n (%)	52 (65.8)	38 (65.5)	14 (66.7)	1.000
TTE, n (%)	57 (72.2)	42 (72.4)	15 (71.4)	1.000
TEE, n (%)	11 (13.9)	6 (10.3)	5 (23.8)	0.246
FUBC, n (%)	75 (94.9)	54 (93.1)	21 (100.0)	0.513
Antifungal therapy, n (%)	43 (54.4)	34 (58.6)	9 (42.9)	0.324
Persistent BSI, n (%)	13 (16.5)	11 (19)	2 (9.5)	0.512
Length of stay, days, median (IQR)	39 (23.5–69.5)	39 (23–69)	36 (26–71)	0.698
30-day death, n (%)	10 (12.7)	6 (10.3)	4 (19)	0.519
Relapse within 90 days, n (%)	23 (29.1)	7 (12.1)	3 (14.3)	1.000

Abbreviations: n = numbers, % = percentage, IQR = interquartile range, VSE = vancomycin-susceptible Enterococcus, VRE = vancomycin-resistant Enterococcus, ICU = intensive care unit, IAI = intra-abdominal infection, CVC = central venous catheter, CVVH = Continuous Venovenous Hemofiltration, TTE = trans-thoracic echocardiography, TEE = trans-oesophageal echocardiography, FUBC = follow-up blood cultures, BSI = bloodstream infection.

**Table 2:** Univariable Cox Regression for 30-day mortality.

Characteristic	HR	95% CI	*p*-Value
Age	1.00	0.95, 1.06	0.9
Sex	1.86	0.52, 6.61	0.3
ICU admission	2.40	0.67, 8.52	0.2
Nosocomial infection	0.88	0.19, 4.15	0.9
Polymicrobial infection	0.29	0.04, 2.30	0.2
Charlson Comorbidity Index	1.03	0.79, 1.35	0.8
Pitt score	2.02	1.33, 3.09	0.001
Pitt score ≥ 2	5.17	1.10, 24.3	0.038
Septic shock	17.1	3.64, 80.8	<0.001
Primary infection	2.28	0.29, 18.1	0.4
IAI	1.40	0.36, 5.40	0.6
CVC/intravascular device related	0.69	0.15, 3.24	0.6
Appropriate empirical therapy	0.24	0.03, 1.86	0.2
Combination empirical therapy	1.52	0.43, 5.40	0.5
Targeted treatment	0.00	0.00, Inf	>0.9
Appropriate treatment withing 24 h	0.91	0.26, 3.22	0.9
Effective antibiotic treatment	0.18	0.01, 2.39	0.2
Targeted combination therapy	2.42	0.63, 9.40	0.2
Source control	0.20	0.05, 0.76	0.018
ID consultation	1.52	0.19, 12.1	0.7
TTE	0.35	0.10, 1.22	0.10
TEE	0.68	0.09, 5.34	0.7
FUBC	25,868,579	0.00, Inf	>0.9
Persistent BSI	1.85	0.48, 7.20	0.4
BSI duration 30	1.00	0.90, 1.10	>0.9
Diabetes mellitus	1.55	0.44, 5.51	0.5
Obesity	1.16	0.15, 9.21	0.9
Chronic obstructive pulmonary disease	1.22	0.15, 9.61	0.9
Ischemic heart disease	0.65	0.14, 3.06	0.6
Hematological cancer	2.61	0.33, 20.6	0.4
Solid cancer	1.20	0.31, 4.65	0.8
Liver cirrhosis	1.09	0.31, 3.78	0.9
Asplenia	0.00	0.00, Inf	>0.9
HIV	0.00	0.00, Inf	>0.9
Liver transplant	0.47	0.14, 1.63	0.2
Kidney transplant	1.50	0.31, 7.12	0.6
Transplant other than liver and kidney	1.99	0.52, 7.72	0.3
Surgery 60 days before admission	0.83	0.21, 3.21	0.8
Total parenteral nutrition	1.36	0.38, 4.82	0.6
Urinary catheter	2.94	0.37, 23.3	0.3
Gram negative rectal colonization	0.84	0.11, 6.65	0.9
eGFR	0.99	0.97, 1.01	0.3
Admission in the previous 3 months	1.20	0.35, 4.16	0.8
Admission in the previous 12 months	0.96	0.25, 3.71	>0.9
CVC	2.90	0.37, 22.9	0.3
CVVH	6.40	1.85, 22.1	0.003
Drainage	1.12	0.29, 4.32	0.9
Vancomycin resistance	1.98	0.56, 7.03	0.3
Ampicillin-resistant strain	73,730,302	0.00, Inf	>0.9
High-level aminoglycoside resistance	0.39	0.10, 1.50	0.2

Abbreviations: BSI = bloodstream infection, CI = confidence interval, CVC = central venous catheter, CVVH = Continuous Venovenous Hemofiltration, eGFR = estimated glomerular filtration rate, FUBC = follow-up blood cultures, HIV = Human Immunodeficiency Virus, HR = Hazard Ratio, ID = infectious diseases, IAI = intra-abdominal infection, ICU = intensive care unit, TEE = trans-oesophageal echocardiography, TTE = trans-thoracic echocardiography. Legend to Table 2. This table reports the results of univariable Cox proportional hazards regression for 30-day mortality in patients with *Enterococcus faecium* bloodstream infection undergoing solid organ transplantation (SOT). Each predictor variable was evaluated individually using a time-dependent covariate structure.

**Table 3:** Multivariable Cox Regression for 30-day mortality.

Characteristic	aHR	95% CI	*p*-Value
VRE	2.07	0.40, 10.6	0.4
Effective antibiotic therapy (time-dependent)	0.31	0.02, 4.11	0.4
CVVH	2.00	0.43, 9.38	0.4
TTE	0.50	0.11, 2.25	0.4
Pitt score ≥ 2	1.33	0.16, 11.2	0.8
Septic shock	11.4	1.63, 79.5	0.014
Source control	0.54	0.10, 3.07	0.5

Abbreviations: aHR = adjusted Hazard Ratio, CI = confidence interval, CVVH = Continuous Venovenous Hemofiltration, TTE = trans-thoracic echocardiography. Legend to Table 3. This table reports the results of the multivariable Cox proportional hazards regression for 30-day mortality in patients with *Enterococcus faecium* bloodstream infection undergoing solid organ transplantation. The analysis was performed with a time-dependent covariate structure.

## Data Availability

The de-identified clinical dataset underlying this article is available from the corresponding author on reasonable request, subject to institutional data sharing agreements.

## Supplementary Materials

The following supporting information can be downloaded at https://www.mdpi.com/article/10.3390/antibiotics15020119/s1. Table S1: Details on resistance phenotype and type and combination of antimicrobial treatments.

## Abbreviations

The following abbreviations are used in this manuscript:

BSI	Bloodstream infection
CI	Confidence interval
CVC	Central venous catheter
CVVH	Continuous Venovenous Hemofiltration
HR	Hazard Ratio
ICU	Intensive care unit
IQR	Interquartile range
SOT	Solid organ transplant
TEE	Trans-oesophageal echocardiogram
TTE	Trans-thoracic echocardiogram
VRE	Vancomycin-resistant Enterococcus
VSE	Vancomycin-susceptible Enterococcus
