# Supplementary material for: Impact of Vancomycin Resistance on 30-Day Mortality in Solid Organ Transplant Recipients with Enterococcus faecium Bloodstream Infections: A Retrospective Cohort Analysis
Source: Antibiotics (Basel). 2026 Jan 26;15(2):119. doi: 10.3390/antibiotics15020119 (PMC12937430; doi:10.3390/antibiotics15020119)
Supplement: Supplementary file 1 [file antibiotics-15-00119-s001.zip › antibiotics-4083045-supplementary.pdf]

Supplementary Table S1. Details on resistance phenotype and type and combination of antimicrobial treatments

Characteristics	Overall, n=79 (%)	VSE, n= 58	VRE, n=21	P value
Linezolid Resistant Strain, n (%)	1 (1.3)	0 (0.0)	1 (4.8)	0.594
Ampicillin Resistant Strain, n (%)	74 (93.7)	53 (91.4)	21 (100)	0.386
High level Aminoglycoside Resistance, n (%)	41 (51.9)	27 (46.6)	14 (66.7)	0.185
Empirical therapy, n (%)	79 (100)	58 (100)	21 (100)	NA
Combination empirical therapy, n (%)	38 (48.1)	27 (46.6)	11 (52.4)	0.839
Empiric beta lactam, n (%)	69 (87.3)	50 (86.2)	19 (90.5)	0.904
Empiric daptomycin, n (%)	8 (10.1)	8 (13.8)	0 (0)	0.170
Empiric glycopeptide, n (%)	13 (16.5)	8 (13.8)	5 (23.8)	0.473
Empiric linezolid, n (%)	8 (10.1)	6 (10.3)	2 (9.5)	1.000
Empiric tigecycline, n (%)	9 (11.4)	7 (12.1)	2 (9.5)	1.000
Appropriate empirical therapy, n (%)	22 (27.8)	20 (34.5)	2 (9.5)	0.057
Appropriate treatment within 24 hours, n (%)	32 (40.5)	26 (44.8)	6 (28.6)	0.298
Targeted treatment, n (%)	76 (96.2)	56 (96.6)	20 (95.2)	1.000
Days to targeted treatment, median (IQR)	2 (0-3)	2 (0-3)	2 (1-4)	0.149
Targeted combination therapy, n (%)	36 (45.6)	25 (43.1)	11 (52.4)	0.634
Daptomycin plus beta lactam, n (%)	12 (15.2)	8 (13.8)	4 (19)	0.826
Daptomycin plus other, n (%)	1 (1.3)	1 (1.7)	0 (0)	1.000
Glycopeptide plus beta lactam, n (%)	9 (11.4)	9 (15.5)	0 (0)	0.129
Linezolid or tedizolid plus beta lactam, n (%)	11 (13.9)	6 (10.3)	5 (23.8)	0.246
Linezolid or tedizolid plus other, n (%)	3 (3.8)	1 (1.7)	2 (9.5)	0.349
Combination with tigecycline, n (%)	4 (5.1)	2 (3.4)	2 (9.5)	0.612
Glycopeptide monotherapy, n (%)	17 (21.5)	17 (29.3)	0 (0)	0.013
Daptomycin monotherapy, n (%)	10 (12.7)	8 (13.8)	2 (9.5)	0.904
Linezolid or tedizolid monotherapy, n (%)	14 (17.7)	6 (10.3)	8 (38.1)	0.012
